# NSP 5a3a: A Potential Novel Cancer Target in Head and Neck Carcinoma

**DOI:** 10.18632/oncotarget.176

**Published:** 2010-09-30

**Authors:** Luca D'agostino, Antonio Giordano

**Affiliations:** ^1^ Sbarro Institute for Cancer Research and Molecular Medicine & Department of Biology, College of Science and Technology Temple University, 1900 North 12th street room 431, Philadelphia PA 19122

**Keywords:** NSP5a3a, P73, Head and Neck Carcinoma, Apoptosis, Cell Cycle

## Abstract

NSP 5a3a along with three other distinct though similar splice variants were initially identified corresponding to locus HCMOGT-1 on chromosome 17p11.2 [1]. Secondary structure analysis of the novel structural protein (NSP) isoforms revealed similarity to Spectrin like proteins containing coiled coil domains [1]. The NSP 5a3a isoform had been found to be highly expressed in-vitro in particular cancer cell lines while very low to un-detectable levels in normal body tissues [1]. Subsequent investigation of this isoform revealed its novel interaction with B23 [2], a multifunctional nucleolar protein involved in ribosome biogenesis, rRNA transcription, mitosis, cell growth control, and apoptosis [3]. Subsequent investigation, elucidated NSP 5a3a's potential involvement in cellular processes such as ribosome biogenesis and rRNA processing by validating NSP 5a3a's novel interaction with B23 and ribonuclear protein hnRNP-L possibly implicating NSP 5a3a's involvement in cellular activities such as RNA metabolism and processing [4]. In this preliminary investigation, we wanted to observe the effect that over-expressing NSP 5a3a may have on cell cycle and its potential application in cancer treatment in aggressive cancers such as head and neck carcinomas. Over-expressed NSP 5a3a in HN30 cells induced a significant degree of apoptosis, an average of a 10.85 fold increase compared to controls 3 days post-transfection. This effect was more significant then the apoptosis observed between Fadu cells over-expressing NSP 5a3a and its controls. Though, the apoptosis induced in the WI38 control cell line showed an average of a 13.2 fold increase between treated and controls comparable to the HN30 cell line 3 days post-transfection. Molecular analysis indentified a novel p73 dependent mechanism independent of p53 and caspase 3 activity through which NSP 5a3a is inducing apoptosis. We propose NSP 5a3a as a potential therapeutic target for site directed cancer treatment in perhaps certain head and neck carcinomas by induction of apoptosis.

## INTRODUCTION

Generally, the process of apoptosis is a critical and essential cellular function allowing regulated turn-over of cells during embryonic development, differentiation, and in response to disease [5-6]. Typically, this complex process which regulates cellular homeostasis is characterized by distinct morphological and biochemical signatures including nuclear condensation, cell shrinkage, formation of apoptotic bodies, cellular blebbing and the externalization of phosphatidylserine [5-6]. Apoptosis can be described as a series of tightly regulated series of molecular events that occur over three distinct stages though involve multiple and in tandem molecular pathways of activation [7]. Initially, there is a signaling stage, which results from various apoptotic stimuli such as DNA damage, growth factors, death receptor activation, and cytokines [7]. Each of these stimuli can respectively activate a subsequent initiator of apoptosis or inhibitor of anti-apoptosis, such as PUMA/Noxa, Bad, Bid, and Bim [7-8]. PUMA and/NOXA as well as Bad can inhibit anti-apoptotic members Bcl-2/Bcl-Xl members where Bid and Bim can activate pro-apoptotic members Bax/Bak [7-8]. This activation of Bax and Bak leads to the cellular commitment stage in apoptosis by which there is an organelle dysfunction of the mitochondria and endoplasmic reticulum characterized along with a change in organelle homeostasis involving calcium levels in turn promoting the release of apoptogenic factors from mitochondria [7-8]. The release of apoptogenic factors such as: of Cytochrome C, SMAC/Diablo, Omi/HtrA2, and AIF (Apoptosis inducing factor) in turn mark the execution stage in which activate further downstream pathways leading to the common end result of cell death [7-8]. Cytochrome C will form a molecular complex with Apaf-1 and caspase 9 leading to formation of an Apoptosome that in turn can activate down-stream effectors such as caspase 3, 6, and 7 [7-8]. SMAC/Diablo along with Omi/HtrA2 can bind to IAPs (inhibitors of apoptosis proteins), such as XIAP and cIAP1, disabling them from inhibiting caspase activation. AIF and endonuclease G can lead to DNA degradation and in turn to cell death independent of caspase activation pathways [7-8].

Mediators of apoptosis and cell cycle arrest such as p53 and its family members p63 and p73 which exhibit p53-like activities which can transactivate many p53 target genes such as PUMA, NOXA, BAX, p21, GAdd45, and MDM2 which are involved as well in apoptosis and cell cycle arrest [9-10]. Typically, when there is DNA damage, hypoxia, and certain oncogenic insult, p53 becomes activated and stabilized by post-translational modifications which in turn can activate the execution of the intrinsic apoptotic pathway through transactivation of pro-apoptotic genes such as BAX, PUMA, NOXA, BIG and Apaf-1, as well as others [11].

Head and Neck squamous cell carcinomas (HNSSCs) usually are responsible for nearly 90% of all cancers originating from head and neck regions [12] such as lip/boccal cavity and various regions associated with the pharynx and larynx [13] making them quite clinically, biologically, and pathologically unique and diverse [14]. It's the eighth most common cancer in United States with approximately 40,000 to some 47,000 new cases diagnosed each year [12, 14] and despite on-going available treatments and combinations involving surgery, chemotherapy, and radiotherapy the prognosis and survival rate of patients with head and neck cell carcinomas is still poor and has not improved significantly [12]. Thus, there has been an urgency to detect potential novel tumor markers for HNSCCs as well as develop new drugs and therapies. Recently, there has been growing evidence suggesting the use of biological therapies or molecular based therapies that target cancer cells possibly increasing these cells sensitivity to radio and chemotherapy treatments involving lower doses or exposures with minimal cytotoxic effects to surrounding healthy cells [15].

NSP 5a3a along with the other three isoforms, NSP 5b3a, 5a3b, and 5b3b were identified having marginal homology to SMC (structural maintenance chromosome), SMC like proteins, DNA repair Sbc proteins, and high similarity to Specc1 and Cytospin A renal carcinoma antigen whose functions are functions are still unknown. The NSP isoforms contain four coiled coil regions characteristic of Spectrin like proteins [2]. These NSP isoforms having features of Spectrin like repeat proteins suggests they may well be part of this protein superfamily as novel members. Generally, Spectrin and Spectrin like proteins have important roles in intracellular structural integrity relating to the cytoskeleton and matrix in both the cytoplasm and nucleus [16-17]. Furthermore, proteins in this superfamily have been found to be involved in intracellular trafficking in secretory pathways [18], maintenance of organelle function, integrity [18], and endocytosis [19], nuclear reassembly in Telophase [20], serving as scaffolding proteins with actin and membrane bound proteins in intracellular signaling pathways [21].

The NSP 5a3a isoform was found to be have differential expression in several cancer cell lines in-vitro though quite high in HT-29, MCF-7, Hela, HN30, Saos-2, CEM, and H23 [2]. NSP 5a3a and NSP 5a3b were shown to interact with a multi-functional nucleophos-phoprotein known as B23 (Numatrin or Nucleophosmin) in Hela cells particularly in mitotic Hela cells. This implied a possible involvement of these two isoforms in mitosis in relation to B23 and possibly other B23 functions too [2]. A subsequent study demonstrated that NSP 5a3a, B23, and hnRNP-L (heterogeneous ribonuclearprotein) interacted with each other in both MCF-7 (breast adenocarcinoma) and MCF-12A (normal breast) cell lines though in a both site-specific and cell specific manner [4]. This novel interaction between NSP 5a3a with B23 and hnRNP-L raised possibilities of NSP 5a3a being involved in RNA metabolism and RNA processing both of which B23 and hnRNP-L are involved [4].

Through over-expression of NSP 5a3a in HN30, Fadu, and WI38 we observed a significant level of apoptosis 3 days post-transfection in HN30 cells (7 to 16.42 fold increase compared to controls) and WI38 cells (11 to 13.45 fold increase compared to c ontrols), both being p53 functional [28-29] though through a p53 independent pathway. In contrast, levels of apoptosis were comparable in the Fadu cell line (1.07 to 1.13 fold decrease) being p53 mutant non-functional [30]. We propose NSP 5a3a as a potential therapeutic target to consider in site-directed delivery to treat cancer in perhaps certain head and neck carcinomas with a similar novel p73 dependent mechanism as the one identified in this study.

## RESULTS

### Morphological and FACS analysis of Fadu over-expressing NSP 5a3a

**Figure 1:**
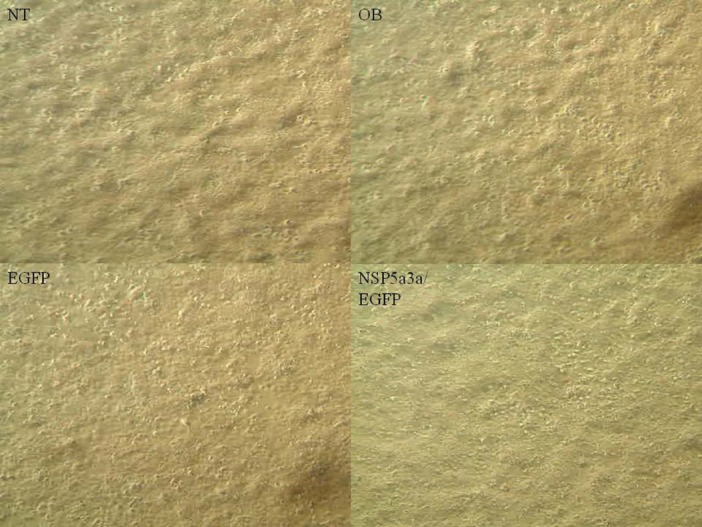
Morphological observation of Fadu cells 3 days post-transfection. NT: non-treated, OB: only buffer, EGFP: only pcDNA3.1/CT-GFP vector, NSP 5a3a/EGFP: pcDNA 3.1/CT-GFP and pcDNA 3.0 NSP 5a3a.

Asynchronous Fadu cells were transfected with pcDNA3.0 NSP 5a3a vector along with controls after which images of the cells were taken three days post-transfection and prepared for FACS analysis to assess the effect of NSP 5a3a over-expression on cell cycle. Images of Fadu cells three days post-transfection revealed that treated cells with NSP 5a3a seemed comparable to the controls (Fig. 1.) Controls and treated cells were seemingly still confluent and with well attached cell populations (Fig. 1.). FACS analysis revealed that there was no significant difference observed between treated cells and controls as well as no significant shifts in cell cycle in treated cells as compared to the controls (Fig. 4.).

**Figure 2:**
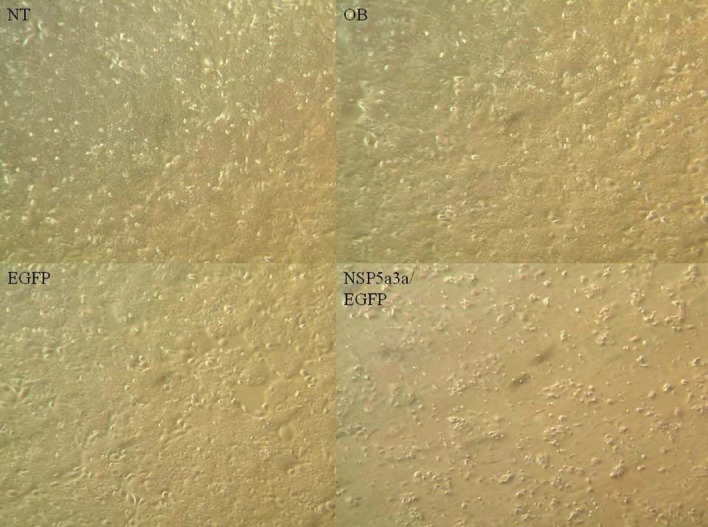
Morphological observation of HN30 cells 3 days post-transfection. NT: non-treated, OB: only buffer, EGFP: only pcDNA3.1/CT-GFP vector, NSP 5a3a/EGFP: pcDNA 3.1/CT-GFP and pcDNA 3.0 NSP 5a3a.

**Figure 3:**
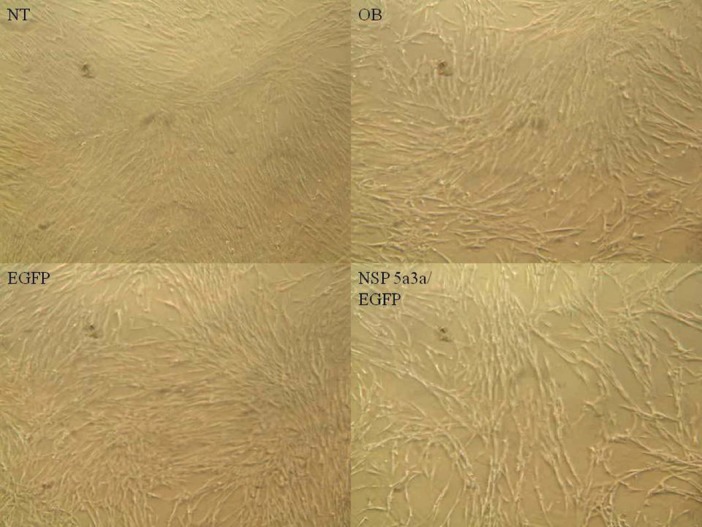
Morphological observation of WI38 cells 3 days post-transfection. NT: non-treated, OB: only buffer, EGFP: only pcDNA3.1/CT-GFP vector, NSP 5a3a/EGFP: pcDNA 3.1/CT-GFP and pcDNA 3.0 NSP 5a3a.

**Figure 4:**
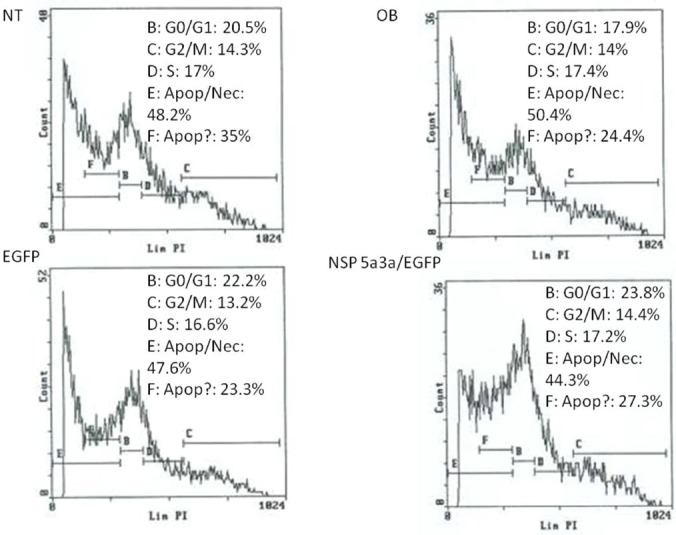
FACS analysis of asynchronous Fadu cells 3 days post-transfection. NT: non-treated, OB: only buffer, EGFP: only pcDNA3.1/CT-GFP vector, NSP 5a3a/EGFP: pcDNA 3.1/CT-GFP and pcDNA 3.0 NSP 5a3a.

### Morphological and FACS analysis of HN30 over-expressing NSP 5a3a

**Figure 5:**
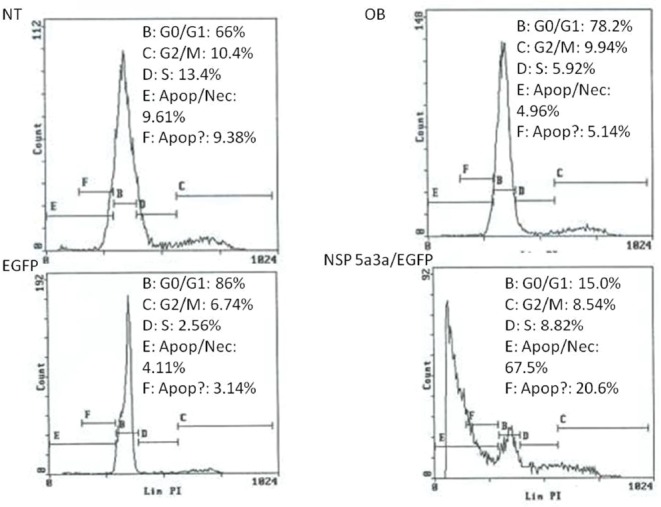
FACS analysis of asynchronous HN30 cells 3 days post-transfection. NT: non-treated, OB: only buffer, EGFP: only pcDNA3.1/CT-GFP vector, NSP 5a3a/EGFP: pcDNA 3.1/CT-GFP and pcDNA 3.0 NSP 5a3a.

Asynchronous HN30 cells were transfected with pcDNA3.0 NSP 5a3a vector along with controls after which images of the cells were taken three days post-transfection and prepared for FACS analysis to assess the effect of NSP 5a3a over-expression on cell cycle. Images of HN30 cells three days post-transfection revealed that treated cells with NSP 5a3a seemed highly apoptotic compared to the controls (Fig. 2.) Controls were seemingly still confluent and well attached cell populations while the treated cells with NSP 5a3a showed a high degree of detached cells in media with very few cells attached (Fig.2). The initial verification of possible apoptosis was verified by FACS analysis which demonstrated that the treated cells over-expressing NSP 5a3a had an average of a10.85 fold increase in apoptosis compared to controls (Fig. 5.). Consequently, there was a 5 fold decrease in treated cells found in G1/G0 while no significant change in the G2/M and S phases was observed in the treated cells as compared to the controls (Fig. 5).

### Morphological and FACS analysis of WI38 over-expressing NSP 5a3a

**Figure 6:**
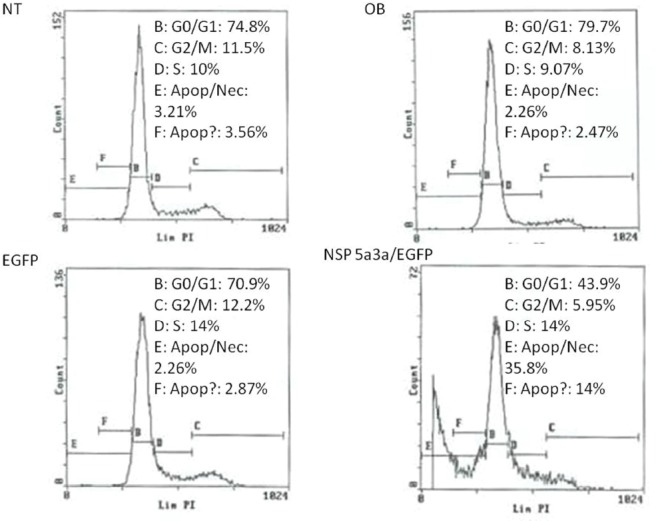
FACS analysis of asynchronous WI38 cells 3 days post-transfection. NT: non-treated, OB: only buffer, EGFP: only pcDNA3.1/CT-GFP vector, NSP 5a3a/EGFP: pcDNA 3.1/CT-GFP and pcDNA 3.0 NSP 5a3a.

Asynchronous WI38 cells were transfected with pcDNA3.0 NSP 5a3a vector along with controls after which images of the cells were taken three days post-transfection and prepared for FACS analysis to assess the effect of NSP 5a3a over-expression on cell cycle. Images of WI38 cells three days post-transfection revealed that treated cells with NSP 5a3a seemed highly apoptotic compared to the controls (Fig. 3.) similar to what was observed in the HN30 cell line. Controls were seemingly still confluent and well attached cell populations while the treated cells with NSP 5a3a showed a high degree of detached cells in media with very few cells attached (Fig.3). Verification of possible apoptosis was confirmed by FACS analysis which demonstrated that the treated cells over-expressing NSP 5a3a had an average of 13 fold increase in apoptosis compared to controls (Fig. 6.). There was approximately an average of a 2 fold decrease in both Go/G1 and G2/M phases in treated as compared to controls though no significant changes observed in S phase (Fig. 6).

### Molecular analysis of NSP 5a3a over-expression in Fadu

**Figure 7:**
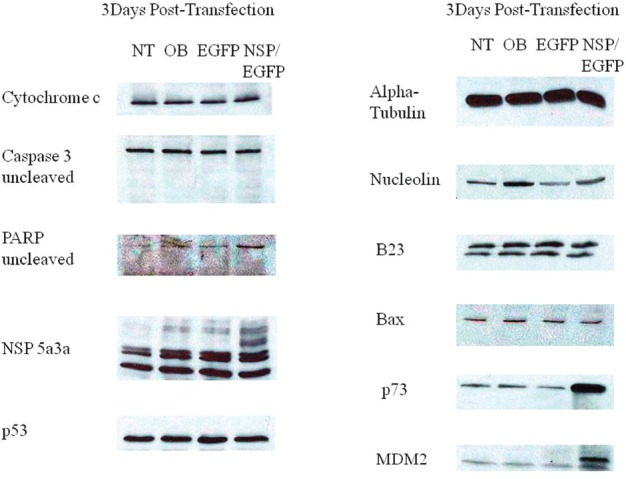
Western Blot analysis of total lysates from asynchronous Fadu cells 3 days post-transfection. NT: non-treated, OB: only buffer, EGFP: only pcDNA3.1/CT-GFP vector, NSP 5a3a/EGFP: pcDNA 3.1/CT-GFP and pcDNA 3.0 NSP 5a3a.

Western blot analysis of total lysates of Fadu cells from three days post-transfection along with controls were analyzed to understand the possible mechanism involved in the absence of an appreciable apoptotic response when over-expressing NSP 5a3a in this particular p53 mutant cell line [30]. There was no apparent significant change in P53, B23, Bax, nucleolin, and Cytochrome c protein levels between treated and the controls. Procaspase 3 levels as well were comparable between controls and treated cells though no cleaved caspase 3 products of 17 and 19 Kda were detected in treated nor in controls. There was no apparent change in un-cleaved Parp 116Kda between treated and controls as also no cleaved Parp product of 89 Kda and 24 Kda was detected either in all conditions. Un-cleaved MDM2 around 90 Kda was not detected in all conditions though there seemed to a substantial increase in the cleaved MDM2 product 60 Kda in the treated cells as compared to the controls. A possible high molecular weight P73 isoform around 140 Kda or above were detected in both treated and controls though treated cells showed a moderate increase in that particular p73 isoform (Fig. 7.). Though, no other p73 isoforms around the 73 Kda range were detected for all conditions. Arf and p21 levels were not detected in both treated and controls (data not shown).

### Molecular analysis of NSP 5a3a over-expression in HN30

**Figure 8:**
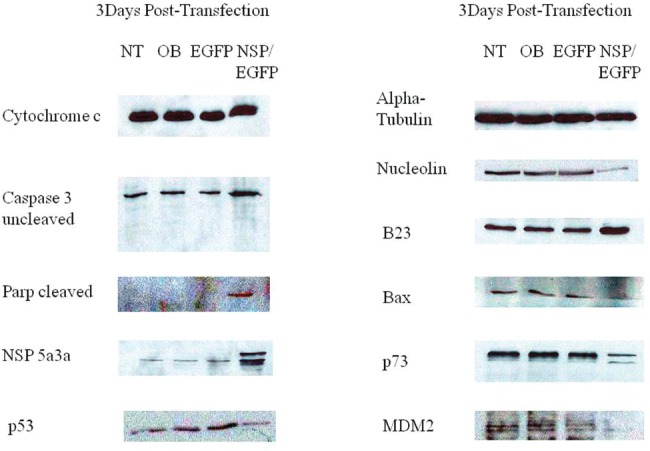
Western Blot analysis of total lysates from asynchronous HN30 cells 3 days post-transfection. NT: non-treated, OB: only buffer, EGFP: only pcDNA3.1/CT-GFP vector, NSP 5a3a/EGFP: pcDNA 3.1/CT-GFP and pcDNA 3.0 NSP 5a3a.

Western blot analysis of total lysates of HN30 cells from three days post-transfection along with controls were analyzed to elucidate the possible pathway that NSP 5a3a may be affecting in order to induce apoptosis in this particular p53 functional cell line [28]. Proteins involved in apoptosis (p53 dependent and independent manner) as well as known to interact with B23 were considered to be studied given the established interaction between NSP 5a3a and B23 in Hela [2] as also in MCF-7 and MCF-12A cells [4]. Bax levels while apparently very low in the cell line showed possibly a slight decrease between treated and controls cells. P53 levels also being very low in all conditions appeared to have possibly a slight decrease in treated cells as compared to the controls. B23 levels showed a slight apparent increase in expression in the treated compared to the controls. Procaspase 3 levels were comparable between controls and treated cells though no cleaved caspase 3 products of 17 and 19 Kda were detected in treated nor in controls. A cleaved Parp product around 89 Kda was detected in treated cells but not in the controls while no un-cleaved Parp around 116 Kda and smaller Parp product of 24 Kda was detected in the treated and controls. Low levels of un-cleaved MDM2 around 90Kda and possibly cleaved around 60Kda were detected in controls though an apparent decrease of both un-cleaved and cleaved were observed in the treated cells. Nucleolin levels while apparently low in the cells line seemed to be decreased in the treated when compared to the controls. Possible high molecular weight P73 isoforms around 140 Kda and above were detected in both treated and controls though treated cells showed a significant decrease in that particular p73 isoform. No p73 isoforms around the 73 Kda range were detected for all conditions. Finally, Cytochrome c levels were comparable between controls and treated (Fig. 8.). Arf and p21 levels were not detected in both treated and controls (data not shown).

### Molecular analysis of NSP 5a3a over-expression in WI38

Western blot analysis of total lysates of WI38 cells from three days post-transfection along with controls were analyzed to elucidate the possible pathway that NSP 5a3a may be affecting in order to induce apoptosis again in this particular p53 functional cell line [29].

There was no apparent significant change in p53, B23, Bax (low levels), Nulceolin (low levels), and Cytochrome c protein levels between treated and controls. P73 protein levels showed a moderate decrease in treated compared to controls again only high molecular weight p73 isoform around or above 140 Kda was detected in all conditions. Procaspase 3 levels were comparable between treated and controls and no cleaved caspase product was detected. Un-cleaved Parp levels were also comparable between treated and controls while no cleaved Parp products were detected in treated and controls. Interestingly, only an apparent decrease in both un-cleaved and cleaved MDM2 while low was observed in treated cells as compared to controls. Arf and p21 levels were not detected in both treated and controls (data not shown).

## DISCUSSION

Apoptosis is critical to maintenance of homeostasis in normal cells also having an important role in tumorigenesis [25-26]. It has been characterized in by specific cellular, biochemical, and molecular events during normal cell turnover, cellular differentiation, organogenesis, and embryonic development as well [27-28]. Typically, apoptosis also known as programmed cell death can be initiated through two main separate pathways that ultimately converge downstream resulting in cell death through the mitochondria: the extrinsic and intrinsic pathway [28].

The extrinsic pathway is usually initiated by external stimuli such as cytokines like TNF, TRAL, and CD95/Fas/Apo-1 families, who bind to specific membrane death receptors, which in turn through an adaptor protein such as Fas which has a death domain, can bind to Procaspase 8 or 10 [8, 29]. This in turn, activates the caspase promoting its cleavage with the subsequent activation/cleavage of caspases 3 and 7. Activation of caspase 3 by caspase 8 can occur by 2 pathways [30]. Caspase 8 activation occurs through self-cleavage and activation which can activate and cleave pro-apoptotic proteins such as BID. This results in cytochrome c release as well as other pro-apoptotic proteins, AIF and Endo G which lead to DNA fragmentation, and antagonists of (IAPs) inhibitors of apoptosis, Smac/Diablo and HtrA2/Omi [8]. The release of cytochrome c also and binding to dATP leads to formation of an apoptosome with Apaf-1 and procaspase 9 which in turns results in caspase 9 activation and subsequent activation of caspase 3. Another manner of caspase 3 activation is by direct activation of caspase 3 by caspase 8 [30]. Finally, caspase 3 activation leads to cleavage of death substrates and cell death.

The intrinsic pathway is usually triggered in response to internal insults such as from oxidative stress, direct DNA damage, oncogenes, hypoxia, and depletion of survival factors [31-32]. Activation of the intrinsic pathway usually occurs through the stabilization and activation of p53, a well known regulator of cell growth arrest and apoptosis [31-32]. This activation leads to transcriptional activation of pro-apoptotic proteins such as Bax and BID as well as modulators of apoptosis, Puma/Noxa [31]. Bax, BID, and BIM can either be involved in formation of pore formation in the mitochondrial membrane leading to release of cytochrome c and other apoptogenic factors as in the extrinsic pathway and also be involved in antagonizing anti-apoptotic proteins, BCl-2 BCL-XL, and BFL1 [31]. Subsequent activation of caspase 9 and down-stream effectors such as caspase 3 and 7 lead to the same cell fate as in the extrinsic pathway.

The investigation of novel molecular targets for use in treatment not only in head and neck carcinomas but other aggressive cancers with typically poor prognosis is crucial regardless of the emergence of new cytotoxic drugs whose toxicity is still high with overall low survival expectancy [15].

**Figure 9:**
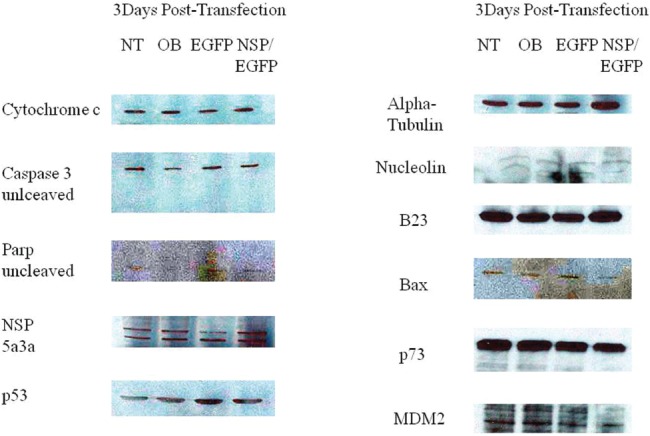
Western Blot analysis of total lysates from asynchronous WI38 cells 3 days post-transfection. NT: non-treated, OB: only buffer, EGFP: only pcDNA3.1/CT-GFP vector, NSP 5a3a/EGFP: pcDNA 3.1/CT-GFP and pcDNA 3.0 NSP 5a3a.

This preliminary study demonstrated that over-expression of NSP 5a3a induced a significant degree of apoptosis in both HN30 and the control cell line WI38 both being p53 functional [22-23] though through a slightly different mechanism in-vitro. The NSP 5a3a isoform seems to be involving two possible nodes of interaction in its induction of apoptosis in the case of HN30 and one for both Fadu and WI38. The node being altered, unique to the HN30 cell line (Fig. 8), is the one involving Nucleolin and Parp, which remained unchanged in the Fadu and WI38 cell lines (Fig. 7 and 9). The other node of interaction shared among all three cell lines is the one involving MDM2 and p73, one that seemingly is up-regulated in the Fadu cell line (Fig. 7) and down-regulated in the HN30 and WI38 cell lines (Fig. 8 and 9).

Though before interpreting the possible mechanisms of action thru which NSP 5a3a is inducing apoptosis in these cell lines let us indicate the commonalities between all three cell lines. In all three cell lines (Fig. 7, 8, 9), there was no significant changes observed between the controls and treated cells for the following proteins which can be involved in apoptosis: p53 levels [31], B23 [33], Bax [34], cytochrome c [35], as well as p14Arf [36] and p21 [37] even though not shown. This indicated to us that the mechanisms of apoptosis were independent of these proteins as well as of caspase 3 since there was the absence of caspase 3 activation [38].

HN30 cells clearly showed the presence of a cleaved Parp product of 89 Kda though its levels were low along with the down-regulation of nucleolin (Fig. 8). Cleavage of Parp usually can be usually accomplished by all caspases in-vitro while only caspase 3 and 7 in-vivo known to be a classical sign of apoptosis in the cells [39]. While no caspase 3 activation was detected for HN30 cells (Fig. 8) pointing to a caspase 3 independent proteolysis of Parp one cannot exclude the possibility of other caspases such as caspase 9 to be involved that can also initiate apoptosis independently of caspase 3 [40].

Parp cleavage has been associated with apoptosis independent of caspase 3 activation as seen in Methotrexate induced apopotosis [41] but also in eleosteric acid induced apopotosis thru AIF release that was independent of Parp activity as well as being Bax and cytochrome c independent apoptosis [42]. It is possible that other caspases may be involved such as caspase 9 as in case of B cell Ag receptor induced apoptosis thru processing of caspase 7 and subsequent Parp cleavage [43]. Typically, if there are signs of apoptosis, there is appearance of the Parp cleavage products 89Kda and 24 Kda fragments [39]. Interestingly, in the case of the HN30, no 24 Kda fragment was detected only the 89-90 Kda fragment. In the cases of Fadu and WI38 ( Fig. 7 and 9) where there is no sign of Parp cleavage neither in the controls nor in the treated cells, there has been indication cited of apoptosis independent of Parp cleavage thus Parp activity [42].

The down-regulation of nucleolin has been associated with initiation of apoptosis in various experimental settings such as its down-regulation in C2C12 cells [44], siRNA knockdown in Hela cells [45], and use of all-trans retinoic acid treatment for induction of apopotsis [46]. Changing levels of nucleolin have also been associated with corresponding changes in Parp levels as well with the possibility that nucleolin may be involved in the processing of Parp and subsequent appearance of the 89-90 Kda Parp fragment associated with apoptosis [47].

Interestingly, we saw a down-regulation in a possible high molecular weight p73 isoform in the treated HN30 cells (Fig. 8). It's been documented that a variety of p73 isoforms exist some being (TA) transactivating forms which can induce apoptosis through activation of p53 target genes while other isoforms being N-terminally truncated are anti-apoptotic [48]. It's possible that the high molecular weight isoform found in our study may be post-translational modification of a p73 isoform since various forms of p73 have been identified around 75, 90 and 140 kDA as found in adult human cortex and hippocampus [49]. It is known that p73 demonstrates p53 like behavior through transcriptional activation of specific genes involved in cell cycle control (e.g. MDM2, Gadd45, PCNA, 14-3-3, p21) and in apoptosis and redox reactions (e.g. PUMA, NOXA, Bax, PIG2-3, PIG 6-8) [50-51]. Though, in p73 induced cell growth arrest and apoptosis, there is up-regulation of some but not all genes regulated by p53 [52]. There is also evidence that the different p73 isoforms possess a quantitative difference in transcriptional activity in the induction of p53 target genes such as p21, Gadd45, MDM2, and p53 [52] thus even leaving the possibility that p73 induces particular genes not regulated by p53 [52].

Normally, TAp73 isoforms (alpha, beta, gamma, delta) are associated with cell cycle arrest and apoptosis though inducing these cellular processes differently depending on the cell type and also activating p53 target genes differently as well [53]. The apoptosis is evidenced by an up-regulation of the particular Tap73 isoform as seen with TAp73 alpha and Tap73 beta [53]. While, on the other hand, the N-terminally p73 isoforms such as delta Np73, ex2p73, and ex2/3p73, which have been found to be highly expressed in many cancers, possess anti-apoptotic activities [54]. Interesting though, there is also evidence that the deltaNp73 isoform can exhibit apoptotic activity when overexpressed [55] as also other deltaNp73 isoforms while targeting p53 specific genes [55]. Given the specificity of the antibody used for p73 in our study we cannot be sure about which p73 isoform is being detected though the antibody was cited to be able to detect all p73 isoforms if present meaning even in the 70 Kda and 55 Kda range corresponding to the TAp73 isoforms (α,β,γ,δ,ε,η,ζ) [54, 56] though we were not able to detect any bands at that height.

Since the antibody in question recognizes the first 80 amino acids of p73, it should be able to detect not only the apoptotic TAp73 isoforms (α,β,γ,δ,ε,η,ζ) [54] if present but also the ex2p73 (α,β,γ, δ,ε,η,ζ) and ex2/3p73 (α,β,γ, δ,ε,η,ζ) isoforms, which are known to be anti-apoptotic [54]. The fact there is an evident decrease of a high molecular weight isoform in both the HN30 treated and the WI38 treated cells, leads us to believe that it may represent post-translational modification of either a TAp73 isoform though one would expect it to be up-regulated in case of apoptosis or that of an anti-apoptotic isoform relating to the ex2p73 or ex2/3p73 isoforms, since one could rationalize a down-regulation of an anti-apoptotic isoform during the occurance of apoptosis though we cannot exclude the possibility of some unknown role of a TAp73 isoform being instead down-regulated in these experimental conditions of apoptosis.

In contrast to HN30 and WI38 treated cells (Fig. 8 and 9), we notice that the Fadu cell line (Fig. 7) instead show an up-regulation of p73 which was still identified in the same height as those for the HN30 and WI38. If we rationalize that the same or similar isoform is being detected in all three cell lines, one could expect this isoform to be an anti-apoptotic form post-translationally modified, since in the Fadu where there was a slight decrease in apoptosis compared to the controls (Fig. 4), we see the isoform being up-regulated while in the HN30 and WI38 it behaves the opposite supporting what was observed (Fig. 5 and 6). This post-translational modification could represent a novel form of modification not yet investigated or previously detected in these cell lines in the context of apoptosis presented.

We see the presence of uncleaved and cleaved MDM2 in all controls for the unstressed HN30 and WI38 cells (Fig. 8 and 9). Though, even so, the Fadu, which seemed to show a natural high degree of apoptosis even in the controls were not caspase 3 dependent. There is a decrease in both the un-cleaved and cleaved MDM2 in the treated for the HN30 and WI30, which seem to accompany the down-regulation of the high molecular weight p73 isoform. This is contrasted in the treated Fadu cells (Fig. 7), which show only the presence of an up-regulated 60Kda MDM2 product that is up-regulated along with an increase in the high molecular weight p73 isoform. It is known that cleavage of MDM2 occurs in apoptosis by means of caspase 3 and even so the 60 Kda fragment usually seen in apoptosis can be seen in unstressed conditions though most likely not involving an apoptosis specific caspase 3[57]. The appearance of the 60Kda fragment usually under these conditions is associated with no Parp activity meaning no Parp cleavage [57].

MDM2 down-regulation has been linked to sensitizing cells to DNA damaging agents thus promoting apoptosis thru p53 pathway though MDM2 can act independently of p53 interacting with E2F family member proteins [58-59] that are known also to induce apoptosis thru p73 apha and beta isoforms [59]. E2F-1 induced apoptosis has been associated with decreased levels of MDM2 levels, no change in levels of Bax nor in p53 though there is evidence of Parp cleavage and caspase activation implicated with caspase 3 [58]. It's known that p73 can transactivate MDM2 in its up-regulation though there is no indication of it down-regulating MDM2 directly being cited and though MDM2 is known to be a negative regulator of p53 [59-60] promoting its degradation though MDM2 does not degrade p73 [59-61].

Nucleolin has been described as negative regulator of MDM2, in which nucleolin over-expression determined decreased levels of HMD2 leading to apoptosis thru a p53 dependent pathway involving up-regulation of p21 as well [62]. This scenario though is not what we observe for the HN30.

Interestingly, when we analyzed NSP 5a3a with a protein motif finder available at http://motif.genome.jp/, four distinct protein signatures associated with apoptosis were detected. One for Tumor necrosis Factor alpha at amino acid sites: 140-158, 154-172, 133-151, and 277-295; Second for Bcl-2 apoptosis regulatory family at amino acid site: 639-667; Third for Daxx protein at amino acid site: 276-330; Fourth for CD97 (Fas) protein at amino acid site: 76-94. TNF-alpha induced apoptosis in Fl5.12 B lymphoma cells involved caspase 9 activation independent of cytochrome c though involved caspase 3 activation downstream [63]. Caspase 9 could be activated by caspase 8 which also can activate caspase 3 as well [63]. Even in endoplasmic reticulum (ER) induced apoptosis, there can be caspase 9 activation independent of cytochrome c though again involving caspase 3 [64]. The fact that we see relatively constant levels of cytochrome c throughout controls and treated in all three cell lines seems to indicate that the apoptosis observed in independent of cytochrome c.

Tumor necrosis factor alpha (TNF-α) is a cytokine known to be involved in both survival pathway via NF-kB or apoptosis through caspase cascade activation [65].

When TNF-α is involved in apoptosis, it binds to tumor necrosis factor receptor type I (TNFRI) which in turns allows the formation of a TRADD complex including FADD and por-caspase 8, this it turn activates caspase 8 and allows the initiation of the death signaling cascade [64, 66]. Caspase 8 can activate other downstream caspases directly as well cleave BID, which in association with BAX and BAK, can cause release of mitochondrial apoptotic factors and further activate the apoptosome and other downstream caspases [63, 66].

TNF-alpha induced has been associated with p73 though typically relating to its up-regulation or over-expression such as thru TAp73 alpha and beta associated with Parp cleavage and caspase 3 activity [67-68]. Thus far, p73 is known to be three distinct pathways leading to apoptosis: one involving over-expression of Tap73alpha leading to Scotin transactivation thus ER stress and apoptosis [69], second involving transactivation of Puma with subsequent translocation of Bax and cytochrome c release [69], and third involving a death receptor pathway in which Tap73 activates death receptors such as CD95 thus leading to caspase activation and cleavage of death substrates (69). The Tap73 activation in the death receptor pathway has been linked also to up-regulation of Fas, a tumor necrosis factor related protein (TNFR) though involving caspase 3 activation [70].

Since there is evidence of caspase 9 dependent but caspase 3 independent pathways [40] as also the implication of caspase 8 activation and cleavage of caspase 9 in death receptor pathways independent of cytochrome c release [71] there may be a novel apoptotic pathway in which NSP 5a3a is involved in its induction. It has been demonstrated that TNF alpha can induce apoptosis thru caspase 8 activation which leads to Bid activation and subsequent cytochrome c release from the mitochondria followed by caspase 9 activation then caspase 3 activation and parp cleavage [66, 72] but also thru caspase 8 activation and direct caspase 3 activation with Parp cleavage [73-74] yet there is also evidence of TNF alpha induced apoptosis independent of caspase 3 activation thru Bak [75].

Fas induced apoptosis typically involves activation of Fas receptor (TNF-R) a tumor necrosis factor member by Fas ligand such as FAsL, member of the tumor necrosis factor cytokine family [76]. This receptor activation leads to recruitment of adaptor proteins such as FADD, Daxx, RIP, FAF-1, and FAP-1 [77]. There is subsequent recruitment of procaspase 8 to the receptor complex where it undergoes self-activation followed by caspase 3 activation thru two distinct manners: one pathway involves caspase 8 cleavage of BID thus triggering cytochrome c release. Cytochrome c release along with procaspase 9 and dATP activates caspase 9 which then activates caspase 3; the second pathway involves the direct activation of caspase 3 by caspase 8 [77]. Though, there is evidence also that Fas/FADD induced apoptosis can also be independent of caspase 3 [78].

Daxx protein is known to be involved in Fas-induced apoptosis by linking the Fas signaling pathway to the JNK pathway through ASK1 activation [79]. Though the role of Daxx being either apoptotic or pro-apoptotic has been under on-going study and discussion [79], it has been found to interact with p73 and function as a transcriptional repressor of p53 and its members including p73 [79].

Even though, further investigation of other caspases as well as other apoptotic proteins should be investigated in NSP 5a3a's induction of apoptosis in head and neck carcinomas as also other cancer cell lines of different origins we can rationalize a possible mechanism. We propose the following hypothetical scenario for the observed apoptosis in the HN30, WI38, and Fadu cell lines. Given the existence of potential protein motifs for TNF-a, CD97, BCL-2, and Daxx in NSP 5a3a, there is possibility that NSP 5a3a may be involved in death receptor mediated apoptosis in a novel manner unseen before in structural proteins with spectrin like repeats and even in SMC proteins which they have both varying degree of homology too. NSP 5a3a may be able to induce apoptosis by possibly activating either the TNF-a or Fas induced apoptotic pathway where in the case of Fas induced apoptosis, it could interact with Daxx influencing thus the regulation of p73 [74], where we could be seeing the down-regulation of an anti-apoptotic isoform both in the HN30 and WI38. NSP 5a3a may be affecting levels of nucleolin in the case of HN30 by possibly affecting one of its transcriptional regulators such as myc [80-81] or post-transcriptional regulators such as hnRNP-L [4]. This in turn would influence the activity and levels of Parp in parallel with nucleolin. The MDM2 levels may be possibly modulated by either a novel dependent or independent manner involving p73 down-regulation.

What we observe may be a completely novel mechanism of apoptosis involving NSP 5a3a, a novel structural protein, acting through a novel p73 dependent manner independent of p53 and independent of caspase 3 activation though possibly acting through other upstream and downstream caspases such caspase 8, 9 and 6, 7 respectively, though seemingly independent of cytochrome c since there was no observed increase in its levels. Even though NSP 5a3a has been cited to have slight to moderate degree of homology to structural maintenance proteins [1], SMC overexpression as with SMC 3 has been associated with transformation and tumorigenesis [82-83] while its down-regulation was associated with apoptosis [84] which is not the case with NSP 5a3a.

Naturally, there must be other apoptotic, anti-apoptotic, and cell cycle proteins involved not yet identified that can explain the difference in how and which molecular nodes are being activated which are seemingly cell-line specific. We demonstrated that NSP 5a3a is involved in the induction of apoptosis by an unknown p73 dependent pathway perhaps not yet indentified involving a structural protein such as NSP 5a3a. Thus, we propose that over-expression of NSP 5a3a could be a feasible means of therapy by induction of apoptosis if used perhaps in a site-directed manner which yet remains to be investigated and determined. Though, we should not exclude testing its applicability and expression not only in other head and neck carcinomas but other cancer cell lines of different origins as well as other normal and primary cell lines too. A broader range of cell line testing and comparison will ultimately give us a better understanding on how NSP 5a3a is inducing apoptosis if so and if there is similarity between cancer cell lines and normal cell lines and what these implications are for a plausible treatment but also its involvement in tumor development and normal biological processes.

## Materials and Methods

### Cell Lines and Tissue Culture

We used following cell lines in this study: Fadu (Head and Neck carcinoma), HN30 (Head and Neck carcinoma), and WI38 (Normal Lung fibroblast). All these cell lines were obtained from ATCC and cultured at conditions recommended by ATCC except for the HN30 cell line which was a kind gift from Dr. George Yoo of the Department of Otolaryngology, Head and Neck Surgery, Wayne State University and Karmanos Cancer Institute, Detroit, Michigan.

### DNA Transfection and Western Blot Analysis

Asynchronous Fadu, HN30, and WI38 cells were seeded in 6 well-plates and transfected and optimized with Fugene HD (Roche) according to the manufacturer's protocol using 2 ug of plasmid DNA per well. Plasmids used for transfection were the following: pcDNA3.1/CT-GFP (invitrogen) and pcDNA 3.0 NSP 5a3a. The NSP 5a3a cDNA had been cloned from a previous work [1]. The pcDNA3.1/CT-GFP vector was used to monitor transfection efficiency for all cell lines in the study.

Fadu, HN30, and WI38 cells at three days post-transfection were harvested mechanically with a scraper, spun and washed twice with PBS 1x after which cells were prepped for western blot analysis. Cell pellets were lysed using a total lysis buffer (50mM Tris-Cl pH 7.4, 5mM EDTA, 250 mM NaCl, 50 mM NaF, .1% Triton X-100, .1 mM Na3VO4, final volume with dH2O) for 15 minutes on ice then for 30 minutes on a rotator at 4 degrees. Lysates were spun down at 13,000 rpm for 15 minutes at 4 degrees after which supernatants were collected and protein concentration was determined by Bradford Assay. A total of 30 ug of total protein was loaded for each sample and separated on 7% SDS-PAGE or 15% gel depending on the molecular weight of the proteins to be separated then followed by transfer onto Whatman Protran Nitrocellulose Transfer Membrane for 1.5 hours at 70 volts at 4 degrees.

Membranes were pre-blocked overnight at 4 degrees in 5% milk buffer TTBS. Next day, membranes were cut and incubated with primary rabbit polyclonal NSP5a3a (Novus) at 1/500, primary mouse monoclonal B23 (Sigma) at 1/500, primary mouse monoclonal MDM2 (SMP14 Santa Cruz) 1/250, primary rabbit polyclonal p73 ( H-79 Santa Cruz) at 1/250, primary mouse monoclonal p53 clone Pab1801 (Santa Cruz) at 1/100, primary mouse monoclonal alpha-tubulin (Invitrogen) at 1/5000, primary mouse mononclonal Bax (2D2 Santa Cruz) at 1/200, primary mouse monoclonal caspase-3 (Cell Signaling) at 1/250, primary mouse monoclonal Cytochrome c (Santa Cruz), primary rabbit polyclonal Parp (Cell Signaling) 1/250 or primary mouse monoclonal Nucleolin (Santa Cruz) at 1//200, primary rabbit polyclonal p21 (BD Biosciences) at 1/500, and primary mouse monoclonal p14 Arf (clone 4c6/4 Santa Cruz) at 1/100 in 5% milk buffer TTBS for 2 hours at RT.

Membranes were then washed 4 times at 15 minute intervals in .1%Tween in PBS 1x then were incubated with either secondary anti-rabbit HRP (Amersham Biosciences) at 1/5000 or secondary anti-mouse HRP (Amersham Biosciences) at 1/5000 in 5% milk buffer for 1 hour at RT. Membranes were then again washed 4 times at 15 minute intervals each and then exposed for 3 minutes to ECL Chemiluminescent Detection Reagent (Perkin Elmer) to be developed on Kodak x-ray film.

### FACS Analysis

Asynchronous Fadu, HN30, and WI38 cells, three days post-transfection were trypsinized, spun for 5 minutes at 1200 rpm and washed in PBS 1x, then fixed in 70% ethanol with 1% FBS overnight at 4°C. Next day cells were spun for 5 minutes at 1200 rpm and washed in PBS 1x twice, afterwards cells were incubated with 300 ul of PBS1x with PI (propidium iodide) and Rnase for 30 minutes at 37°C covered and taken to FACS analysis at Wistar Institute, Philadelphia, Pa.

### Light Microscopy

Asynchronous Fadu, HN30, and WI38 cells, three days post-transfection were observed for morphological changes using Zeiss Axiovert 25 microscope with 10x PH1 Zeiis objective.
